# Editorial: Transgender health: exploring diversity in the endocrine field

**DOI:** 10.3389/fendo.2024.1385262

**Published:** 2024-03-01

**Authors:** Adriana De Sousa Lages

**Affiliations:** ^1^ Endocrinology Department, Braga Hospital, Unidade Local de Saúde (ULS) de Braga, Braga, Portugal; ^2^ Faculty of Medicine, University of Coimbra, Coimbra, Portugal

**Keywords:** transgender, transsexualism, gender dysphoria, gender identity, human rights, sexual minorities and health disparities

Transgender care represents a growing field of interest for different health professionals, as there is an increasing number of individuals who identify as transgender and gender diverse (TGD) seeking care from multidisciplinary teams around the world.

Historically, gender minority stress, as well as financial insecurity, have been associated with significant vulnerability and explain a recurrent delay and avoidance in obtaining access to personalized care for TGD people (1).

In addition, the published research exposes some weaknesses, since most of the studies are empirical studies with small sample sizes and suboptimal design, with subsequent low-quality overall evidence (2).

This Research Topic focuses on improving access to high-quality clinical research, the exchange of practices between health professionals dedicated to transgender care, as well as an opportunity to reflect on the best strategies for improving care and well-being in a responsible manner.

The main aim of this special Research Topic is therefore to explore new insights in the field of transgender health, with the goal of discussing relevant resources and research advances in transgender health practices. Along these lines, the following three papers have been included in the current Research Topic.

The first article in this Research Topic (Jasuja et al.) focused on the concordance between data from clinical practice and the recommendations of the guidelines on gender-affirming hormone therapy (GAHT) as a quality measure for evaluating available care.

The authors report special clinical characteristics of a Veterans Health Administration (VHA) sample, as well as a low rate of complications associated with both feminizing and masculinizing therapy, even among older patients and those with a higher rate of comorbidities, which reassures the safety of GAHT. Nevertheless, the authors stress the need to improve cancer screening and bone health assessment in the TGD population, particularly in those seeking masculinizing therapy.

The progressive importance of precision medicine in the pharmacological approach offered to the TGD population is highlighted in an article by Sehgal. The paper offers a robust narrative review of the pharmacodynamic differences between available drugs and the potential pharmacogenetic influence of currently available pharmacotherapy. Comparative data on hormone therapy in different clinical contexts (dose and posology) also highlight the specificities of the TGD population.

Polymorphisms in the estrogen receptor, especially estrogen receptor 1 (ESR1), may influence the response to estrogen therapy, with a particular clinical impact on bone mineral density during feminizing therapy, although data on the impact of therapeutic response is scarce.

The relationship between the morbidities of the transgender population and the quantification of health burden is explored by Hughto et al., using data from 9,975 transgender individuals.

This work reaffirms the disparities in health diagnosis, especially in the aging trans female and non-binary population, underlining the role of stigma and its stress-related impact on transgender individuals.

The authors focus on the allostatic burden in the TGD population, such as the cumulative burden of chronic stress and life events, and adverse health outcomes (including metabolic, mental health and neoplastic diseases) compared to the cisgender population.

These data contribute to a greater awareness of the care provided to transgender people, identifying potential risk factors for comorbidities, ensuring that transgender individuals receive the necessary information to give their informed consent when THAG or any other gender-affirming procedure is proposed, and highlighting the need for individualized follow-up of this population throughout life.

## Author contributions

AL: Conceptualization, Validation, Writing – original draft, Writing – review & editing.
